# Bleeding parastomal varices in a case of decompensated cirrhosis with tubercular abdominal cocoon: endoscopic ultrasound-guided angioembolization with coil and glue to the rescue

**DOI:** 10.1055/a-2316-0994

**Published:** 2024-05-29

**Authors:** Jahnvi Dhar, N. Pardhu Bharath, Gaurav Mahajan, Harish Bhujade, Pankaj Gupta, Antonio Facciorusso, Jayanta Samanta

**Affiliations:** 1 Department of Gastroenterology, Post Graduate Institute of Medical Education and Research, Chandigarh, India; 2 Department of Radiodiagnosis and Imaging, Post Graduate Institute of Medical Education and Research, Chandigarh, India; 3 Department of Medical and Surgical Sciences, University of Foggia, Foggia, Italy

A 52-year-old man with a 5-year history of alcohol-related decompensated cirrhosis presented with a stomal bleed, and postural symptoms for 15 days. He was diagnosed with abdominal cocoon with intestinal obstruction 2 years previously, for which he underwent ileostomy and received modified antitubercular therapy.

On admission, his vital signs were stable and investigations revealed low hemoglobin (4.2 gm/dL), raised bilirubin (4.3 mg/dL), with normal creatinine. After initial resuscitation with blood transfusions, he underwent esophagogastroduodenoscopy, which revealed obliterated esophageal varices. Computed tomography with angiography (CTA) showed features of cirrhosis, abdominal cocoon, and multiple varicosities at the stomal site ( Fig. 1 ). Stoma site endoscopy revealed a normal efferent limb, hyperemia erosions in the afferent limb, but no definite bleeding site or visible varix ( Fig. 2 ). Radial endoscopic ultrasound (EUS) through the stoma showed two vascular channels with Doppler flow, suggestive of varices ( Fig. 3 ). As the patient was unsuitable for transjugular intrahepatic portosystemic shunt (TIPS) owing to previous episodes of hepatic encephalopathy, a multidisciplinary team discussion took place, and EUS-guided angioembolization was planned for stomal varices management.

**Fig. 1:**
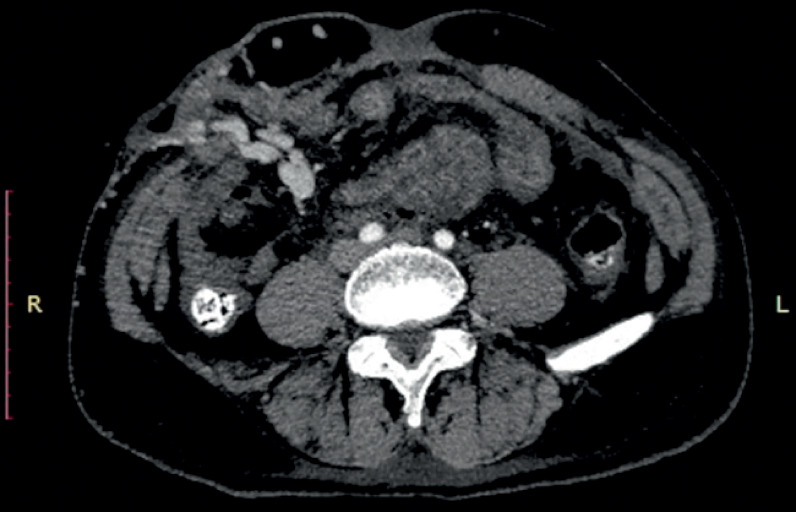
Computed tomography with angiography revealed cirrhosis of the liver (not shown), clustered small-bowel loops encapsulated in a thick membrane-like sac in the mid abdomen (abdominal cocoon), and mild ascites, with two dilated vascular channels (varicosities), going toward the stoma site, involving the bowel wall of the ileostomy.

**Fig. 2:**
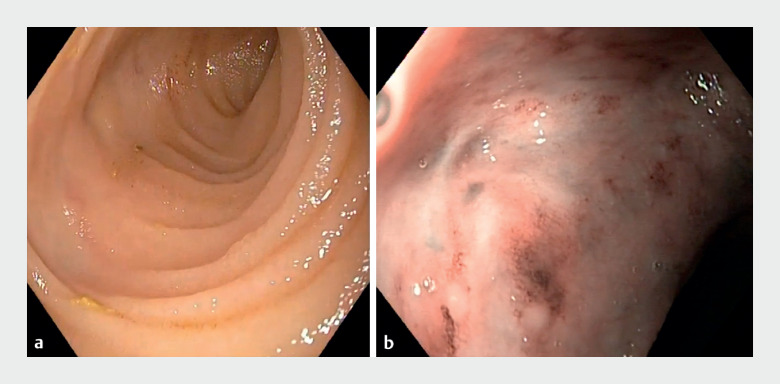
Stoma site endoscopy. **a** Normal efferent limb. **b** Afferent limb showing hyperemia erosions but no definite bleeding site or visible varix.

**Fig. 3:**
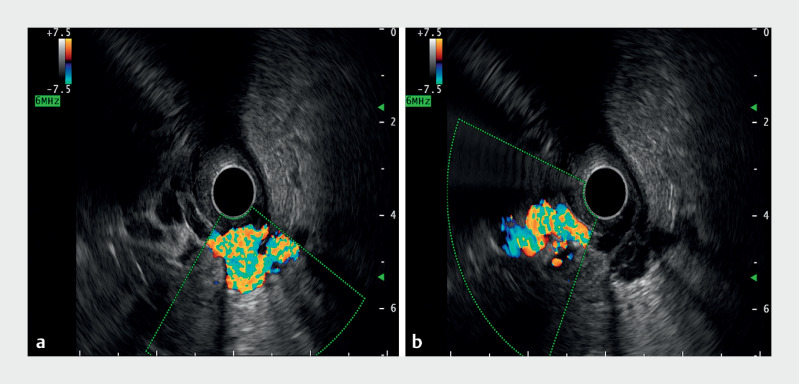
Radial endoscopic ultrasound through the stoma site revealed two vascular channels with positive Doppler flow, suggestive of varices.

Varices were localized using a linear echoendoscope (GIF UCT180; Olympus, Tokyo, Japan), punctured with a 19-G needle (EZ Shot3 Plus; Olympus, Tokyo, Japan), and the position confirmed with blood aspiration. Angioembolization of the varix was performed by deploying a Nester coil (10 mm × 7 cm; Cook Medical, Bloomington, Indiana, USA) followed by injection of 2 mL cyanoacrylate glue, and obliteration was confirmed using Doppler flow; the varix feeder vessel was similarly treated ( Video 1 ).

Endoscopic ultrasound-guided angioembolization using coil and cyanoacrylate glue of bleeding stomal varices, in a diagnosed case of decompensated cirrhosis with tubercular abdominal cocoon.Video 1

At 1-year follow-up, there were no further episodes of stomal bleeding, and hemoglobin had increased to 9.2 gm/dL. Repeat CTA ( Fig. 4 ) and EUS with Doppler ( Fig. 5 ) revealed obliterated stomal varices (no color flow on Doppler) with coils noted in situ.

**Fig. 4:**
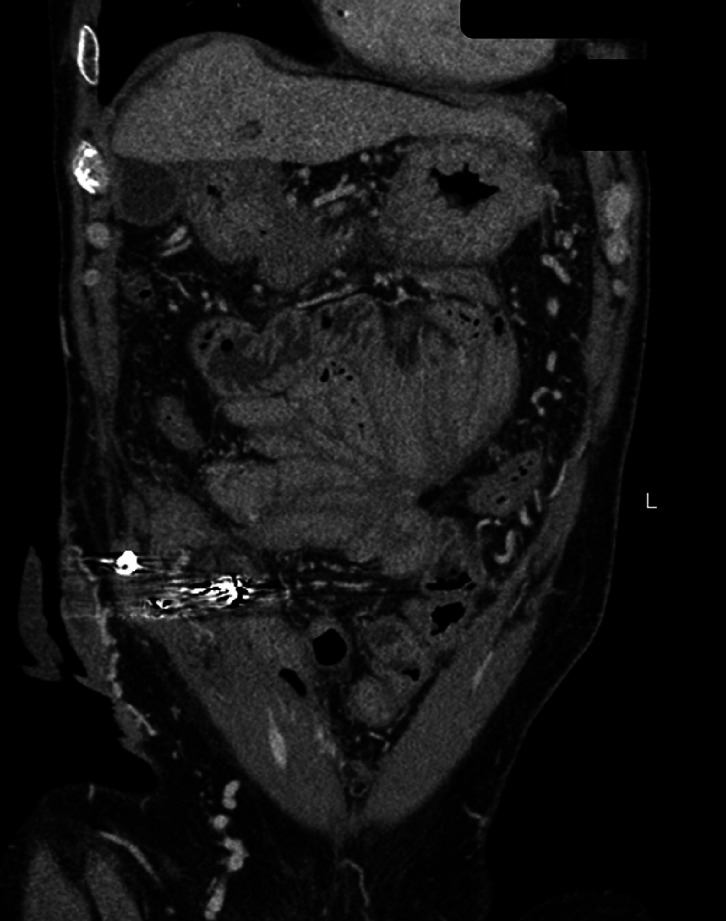
Follow-up computed tomography with angiography revealed obliterated stomal varices with coil noted in situ; also noted were underlying features of liver cirrhosis and mid-abdomen tubercular cocoon.

**Fig. 5:**
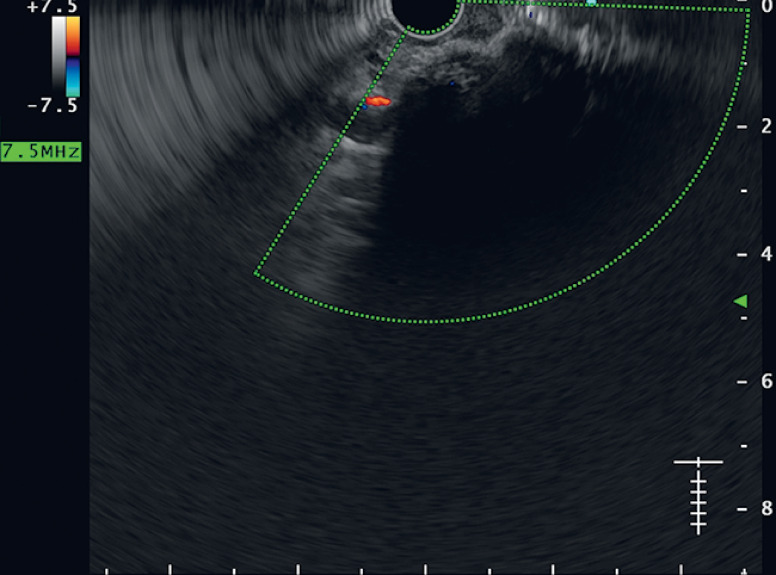
Follow-up endoscopic ultrasound showed obliterated vascular channels (stomal varices), with coils in situ, and no color flow on Doppler.

Bleeding stomal varices account for only 5% of bleeding ectopic varices (1%–5% of all cases) 1 and are a source of great morbidity (13%) and mortality (3%–4%) 2 3 . Our index case was ineligible for TIPS and could not afford liver transplantation. EUS-guided angioembolization allows localization of varices and perforator veins, direct delivery of coils and glue into the varix, and confirmation of obliteration of flow using Doppler 2 3 4 5 , making it a safe and effective modality for management of stomal varices, as shown in our index case.

Endoscopy_UCTN_Code_TTT_1AS_2AG

## Notes

Endoscopy E-Videos https://eref.thieme.de/e-videos
*E-Videos* is an open access online section of the journal *Endoscopy* , reporting on interesting cases and new techniques in gastroenterological endoscopy. All papers include a high-quality video and are published with a Creative Commons CC-BY license. Endoscopy E-Videos qualify for HINARI discounts and waivers and eligibility is automatically checked during the submission process. We grant 100% waivers to articles whose corresponding authors are based in Group A countries and 50% waivers to those who are based in Group B countries as classified by Research4Life (see: https://www.research4life.org/access/eligibility/ ). This section has its own submission website at https://mc.manuscriptcentral.com/e-videos .
